# Supplementary material for: Consistent patterns of common species across tropical tree communities
Source: Nature. 2024 Jan 10;625(7996):728–34. doi: 10.1038/s41586-023-06820-z (PMC10808064; doi:10.1038/s41586-023-06820-z)
Supplement: Supplementary file 1 — Reporting Summary [file 41586_2023_6820_MOESM1_ESM.pdf]

nature portfolio

Corresponding author(s): Declan L M Cooper

Last updated by author(s): Sep 8, 2023

Reporting Summary

Nature Portfolio wishes to improve the reproducibility of the work that we publish. This form provides structure for consistency and transparency in reporting. For further information on Nature Portfolio policies, see our Editorial Policies and the Editorial Policy Checklist.

Statistics

For all statistical analyses, confirm that the following items are present in the figure legend, table legend, main text, or Methods section.

n/a	Confirmed	
☐	☒	The exact sample size (n) for each experimental group/condition, given as a discrete number and unit of measurement
☐	☒	A statement on whether measurements were taken from distinct samples or whether the same sample was measured repeatedly
☒	☐	The statistical test(s) used AND whether they are one- or two-sided<br>*Only common tests should be described solely by name; describe more complex techniques in the Methods section.*
☐	☒	A description of all covariates tested
☐	☒	A description of any assumptions or corrections, such as tests of normality and adjustment for multiple comparisons
☐	☒	A full description of the statistical parameters including central tendency (e.g. means) or other basic estimates (e.g. regression coefficient) AND variation (e.g. standard deviation) or associated estimates of uncertainty (e.g. confidence intervals)
☒	☐	For null hypothesis testing, the test statistic (e.g. F, t, r) with confidence intervals, effect sizes, degrees of freedom and P value noted<br>*Give P values as exact values whenever suitable.*
☒	☐	For Bayesian analysis, information on the choice of priors and Markov chain Monte Carlo settings
☒	☐	For hierarchical and complex designs, identification of the appropriate level for tests and full reporting of outcomes
☒	☐	Estimates of effect sizes (e.g. Cohen's d, Pearson's r), indicating how they were calculated

Our web collection on statistics for biologists contains articles on many of the points above.

Software and code

Policy information about availability of computer code

Data collection	No software was used for data collection.
Data analysis	The custom R code (version 4.3.1) used to run the analyses and produce the figures is available on GitHub with the identifier https://github.com/declancooper/CommonSpecies2022.git

For manuscripts utilizing custom algorithms or software that are central to the research but not yet described in published literature, software must be made available to editors and reviewers. We strongly encourage code deposition in a community repository (e.g. GitHub). See the Nature Portfolio guidelines for submitting code & software for further information.

Data

Policy information about availability of data

All manuscripts must include a data availability statement. This statement should provide the following information, where applicable:

- Accession codes, unique identifiers, or web links for publicly available datasets
- A description of any restrictions on data availability
- For clinical datasets or third party data, please ensure that the statement adheres to our policy

The species abundance data that support the findings of this study are available from https://doi.org/10.6084/m9.figshare.21670883. WorldClim bioclimatic data are available from https://www.worldclim.org/data/bioclim.html.

Human research participants

Policy information about studies involving human research participants and Sex and Gender in Research.

Reporting on sex and gender	NA
Population characteristics	NA
Recruitment	NA
Ethics oversight	NA

Note that full information on the approval of the study protocol must also be provided in the manuscript.

Field-specific reporting

Please select the one below that is the best fit for your research. If you are not sure, read the appropriate sections before making your selection.

☒ Life sciences ☐ Behavioural & social sciences ☐ Ecological, evolutionary & environmental sciences

For a reference copy of the document with all sections, see nature.com/documents/nr-reporting-summary-flat.pdf

Life sciences study design

All studies must disclose on these points even when the disclosure is negative.

Sample size	No sample size calculation was performed. We selected all available plots meeting the following criteria: forest inventory plots ≥0.2 ha in size, situated in structurally intact (no logging or fire), closed canopy (not dry forest or savanna) tropical forest, with enumeration of all stems ≥ 10 cm diameter, in which ≥ 80% of stems are identified to the species level. These criteria allow direct comparisons to be made with results from previous studies investigating common species abundances in Amazonia. The major tropical forest regions of Amazonia, Africa, and Southeast Asia are adequately represented. This is the largest dataset of repeatedly measured plots ever used to calculate long-term trends in African forest carbon dynamics.
Data exclusions	Plots not meeting the following criteria were excluded from analysis: forest inventory plots ≥0.2 ha in size, situated in structurally intact (no logging or fire), closed canopy (not dry forest or savanna) tropical forest, with enumeration of all stems ≥ 10 cm diameter, in which ≥ 80% of stems are identified to the species level. These conditions were pre-established in line with previous studies.
Replication	Replication of repeated random subsampling 200 times ensured that derived results were stable and reproducible.
Randomization	Forest inventory data were partitioned into the the Amazonia, Africa, and Southeast Asia study regions by location. In all of the analyses, sampling and sub-sampling by plots and by trees within plots was done completely randomly within regions.
Blinding	Blinding is not relevant to this study.

Reporting for specific materials, systems and methods

We require information from authors about some types of materials, experimental systems and methods used in many studies. Here, indicate whether each material, system or method listed is relevant to your study. If you are not sure if a list item applies to your research, read the appropriate section before selecting a response.

Materials & experimental systems

n/a	Involved in the study	
☒	☐	Antibodies
☒	☐	Eukaryotic cell lines
☒	☐	Palaeontology and archaeology
☒	☐	Animals and other organisms
☒	☐	Clinical data
☒	☐	Dual use research of concern

Methods

n/a	Involved in the study	
☒	☐	ChIP-seq
☒	☐	Flow cytometry
☒	☐	MRI-based neuroimaging
